# Correction: What is the impact of the COVID-19 pandemic on emergency medicine residency training: an observational study

**DOI:** 10.1186/s12909-022-03786-w

**Published:** 2022-10-18

**Authors:** Hsiang-Yun Lo, Shen-Che Lin, Chung-Hsien Chaou, Yu-Che Chang, Chip-Jin Ng, Shou-Yen Chen

**Affiliations:** 1 Department of Emergency Medicine, Chang Gung Memorial Hospital, Chang Gung University College of Medicine, No. 5, Fuxing St., Guishan Dist, 333 Taoyuan City, Taiwan, Republic of China; 2 The Institute of Health Policy and Management, National Taiwan University, No. 17, Xu-Zhou Road, 100 Taipei, Taiwan; 3 Graduate Institute of Clinical Medical Sciences, Division of Medical Education, College of Medicine, Chang Gung University, No.259, Wenhua 1st Rd.,Guishan Dist, 333 Taoyuan City, Taiwan

Correction: BMC Medical Education (2020) 20:348

10.1186/s12909-020-02267-2

Following publication of the original article [1], the authors informed us that would like to delete the **Additional file 1** because it has improper information.

The original article has been corrected.
